# Chronic Fatigue Syndrome and Chronic Widespread Pain in Adolescence: Population Birth Cohort Study

**DOI:** 10.1016/j.jpain.2016.10.016

**Published:** 2017-03

**Authors:** Tom Norris, Kevin Deere, Jon H. Tobias, Esther Crawley

**Affiliations:** ∗ Centre for Child and Adolescent Health, School of Social and Community Medicine, Bristol, United Kingdom; † Musculoskeletal Research Unit, School of Clinical Sciences, Bristol, United Kingdom

**Keywords:** Chronic fatigue syndrome, chronic widespread pain, ALSPAC

## Abstract

Although many studies have investigated the overlap between pain phenotypes and chronic fatigue syndrome (CFS) in adults, little is known about the relationship between these conditions in adolescents. The study's aim was therefore to identify whether a relationship exists between chronic widespread pain (CWP) and CFS in adolescents and investigate whether the two share common associations with a set of covariates. A questionnaire was administered to offspring of the Avon Longitudinal Study of Parents and Children (ALSPAC) at age 17, asking about site, duration, and pain intensity, from which participants with CWP were identified. At the same research clinic, a computer-based Revised Clinical Interview Schedule was filled out, from which a classification of CFS was obtained. The relationship between selected covariates and CFS and CWP was investigated using a variety of logistic, ordinal logistic, and multinomial regressions. We identified 3,214 adolescents with complete data for all outcomes and covariates. There were 82 (2.6%) individuals classified as CFS and 145 (4.5%) as CWP. A classification of CFS resulted in an increased likelihood of having CWP (odds ratio = 3.87; 95% confidence interval, 2.05–7.31). Female adolescents were approximately twice as likely to have CFS or CWP, with multinomial regression revealing a greater sex effect for CWP compared with CFS. Those with exclusive CFS were more likely to report higher levels of pain and greater effect of pain compared with those without CFS, although associations attenuated to the null after adjustment for covariates, which did not occur in those with exclusive CWP. Multinomial regression revealed that relative to having neither CFS nor CWP, a 1-unit increase in the depression and anxiety scales increased the risk of having exclusive CFS and, to a greater extent, the risk of having comorbid CFS and CWP, but not exclusive CWP, which was only related to anxiety.

**Perspective:**

In this cohort, 14.6% of adolescents with CFS have comorbid CWP. The likely greater proportion of more mild cases observed in this epidemiological study means that prevalence of overlap may be underestimated compared with those attending specialist services. Clinicians should be aware of the overlap between the 2 conditions and carefully consider treatment options offered.

## Highlights

• 14.6% of adolescents with chronic fatigue syndrome (CFS) had chronic widespread pain (CWP).
• Female adolescents were twice as likely to have CFS or CWP.
• Exclusive CFS (vs non-CFS) was associated with higher pain and greater effect of pain.
• This association attenuated after covariate adjustment (depression, anxiety, obesity).
• Higher depressive/anxiety symptoms were linked with higher odds of comorbid CFS & CWP.

Pain is a common comorbidity in adults with chronic fatigue syndrome (CFS; also termed myalgic encephalomyelitis). Indeed, 5 of the 8 and 4 of the 12 accompanying symptoms required to diagnose CFS according to the Centers for Disease Control and Prevention and National Institute for Health and Care Excellence (NICE) criteria respectively, reflect pain^25^, ^39^ (see Supplementary File 1 for the diagnostic criteria used for the classification of CFS and fibromyalgia). Acknowledging this overlap between CFS and pain phenotypes (eg, fibromyalgia, chronic widespread pain [CWP], chronic regional pain), there is phenotypic variation between the sets of conditions, with notable immunological^55^, ^56^ and autonomic^37^ differences observed between the two, as well as discordant patterns of brain activity.^18^, ^59^

Understanding the epidemiology of CFS and the overlap with pain phenotypes (eg, CWP) is important to develop treatment approaches for those with CFS as well as severe pain.^41^ The presence of severe pain in adults with CFS is associated with a worse outcome with cognitive-behavioral therapy^11^, ^29^ suggesting a specific intervention to target pain may improve outcome in this group.^41^ However, without proper understanding of the relatedness of these conditions, this often coming via large epidemiological studies, designing an effective intervention study is difficult.

Adolescents with CFS have levels of pain similar to that of adults with CFS.^16^ Twenty percent of adolescents with CFS experience severe pain (>75 of 100 on the visual analog scale).^16^ Compared with control participants, they report lower pressure pain thresholds,^61^ a greater pain severity, and a greater effect of pain, with the greatest effect occurring at school and for ‘general activity.’^68^ Although there is much literature on the relationship between CFS and pain phenotypes in adults,^1^, ^10^, ^42^, ^71^ little is known about adolescent populations, or the overlap between the 2 conditions.^40^, ^47^, ^52^, ^70^

This study aimed to investigate the relationship between one of these pain phenotypes, CWP, and CFS in adolescents. In particular, we aimed to describe the similarities and differences between CWP and CFS, examine the prevalence of the co-occurrence of these 2 conditions, understand how a classification of CFS or CWP affects the interpretation of the effects of pain (pain intensity, pain interference, and change in behavior), and investigate the association between these and other comorbidities (eg, depression, anxiety, and obesity). Understanding the relationship between and CFS and CWP is important in itself, but any finding could also have relevant implications for juvenile fibromyalgia (because the 2 conditions share similar diagnostic criteria: pain at multiple affected sites, lasting longer than 3 months, and of high intensity).^35^, ^50^, ^54^ The diagnostic criterion for fibromyalgia also includes localized areas of tenderness, which is not included in the CWP diagnosis.

We have previously shown in a large United Kingdom birth cohort, that the prevalence of CFS during adolescence increases from approximately 1.47%^22^ at 13 years to 2.99% at 18 years (Norris, 2016 unpublished data). Within the same cohort, we have also examined the prevalence of musculoskeletal pain phenotypes during adolescence and found that the prevalence of chronic regional pain and CWP at aged 17 years was 4.7% and 4.3%, respectively (n = 3,376).^23^ Our hypothesis was that CFS and CWP would share common associations, on the basis of findings from separate studies that have revealed common covariates (eg, obesity,^23^, ^43^, ^44^ depression,^4^, ^7^ anxiety,^27^, ^66^ socioeconomic status,^5^, ^22^ and female sex^5^, ^13^, ^15^, ^22^, ^67^) associated with CFS as well as various pain phenotypes, including CWP. To the authors’ knowledge, no study in adolescence has sought to compare those experiencing exclusive CFS, exclusive CWP, or both, and identify covariates for these conditions, in the same population. As such, we sought to investigate the relationship between CWP and CFS and a range of covariates, after taking into account the overlap which exists between the two, within a sample of adolescents participating in the Avon Longitudinal Study of Parents and Children (ALSPAC).

## Methods

### Study Population

The ALSPAC is a geographically based United Kingdom cohort that recruited pregnant women living in the former county of Avon (Southwest England) with an expected delivery date of April 1, 1991 through to December 31, 1992.^8^ A total of 14,541 pregnant women were enrolled with 14,062 children born. The children have been followed-up regularly since birth with postal questionnaires for children and their parents, clinical assessments, and the collection of biological samples (please note that the study Web site contains details of all the data that are available through a fully searchable data dictionary: www.bris.ac.uk/alspac/researchers/data-access/data-dictionary/). This study is on the basis of the individuals who completed the pain questionnaire and the computer-based clinical interview at the age 17 research clinic (n = 5,217). Response rates for the assessments from 4 weeks to 18 years have been described.^8^ The ALSPAC aims to increase the completeness of data and better characterize nonresponders using data linkage to routinely collected data sources, which will also provide further insights into participation biases.^8^ Ethical approval was obtained from the ALSPAC Law and Ethics Committee. Parental consent and child's assent was obtained for all measures.

### Pain Questionnaire

A structured pain questionnaire was administered at the 17-year clinic (mean age at attendance 17 years, 10 months), assembled from domains and scales previously validated in United Kingdom populations.^23^ It was given to all participants that attended the 17-year clinic where it was completed on the day of attendance, or returned by post if this was not possible. Participants were asked to shade in the site of pain they had on a manikin and indicate whether the pain had started within the past 3 months or more than 3 months ago (maximum 6 months ago).^32^ CWP was comprised of pain longer than 3 months in duration, axial pain, and upper right quadrant pain plus lower left quadrant pain, or upper left quadrant pain plus lower right quadrant pain, assessed according to the shading of the pain manikin by the participants.^23^ This variable was on the basis of the diagnostic criteria for fibromyalgia of pain on both sides of the body, above and below the waist, and in the axial skeleton, which has been present for 3 months or longer. The diagnostic criterion for fibromyalgia also includes localized areas of tenderness, which were not assessed on the pain questionnaire. Separate to the questions contributing to the classification of CWP, participants were asked about headaches and abdominal pain during the past month on a troublesome scale ranging from no pain, not at all troublesome, slightly troublesome, moderately troublesome, very troublesome, to extremely troublesome.^46^

The pain questionnaire also assessed the participants’ experience of pain on a 1 to 10 to scale where 1 was equal to “no pain” and 10 was equal to “pain as bad as could be.” The questions included how intense was the worst pain in the past 6 months and how intense was the pain on average in the past 6 months. A similar 1 to 10 scale was used to assess how much their pain had interfered with daily activities in the past 6 months and how their pain has changed their ability to take part in recreational, social, and family activities (disruption to social activities).^65^

### CFS

At the same clinic, participants completed a computer-based Revised Clinical Interview Schedule (CIS-R).^30^ We used established criteria^39^ to define CFS. Participants needed to be disabled by their fatigue, to have fatigue that lasted >6 months, and required 1 additional symptom. In addition, we excluded those with other explanations for their fatigue.

Participants were classified as CFS if they indicated that they had been getting tired or had been lacking in energy during the past month and then responded ‘yes’ to >2 of the following 4 items: 1) felt tired or lacking in energy for 4 days or more in the past 7 days, 2) felt tired or lacking in energy for more than 3 hours in total on any day in the past 7 days, 3) felt so tired or lacking in energy that they had to push themselves to get things done on 1 or more occasion in the past 7 days, and 4) felt tired or lacking in energy when doing things they enjoy in the past 7 days. Participants were classified as not chronically fatigued if: the tiredness or lack of energy had lasted for <6 months, the adolescent thought it was due to exercise or medication, the adolescent felt better after resting, if daily activities were not impaired, or if exercise did not make them feel exhausted the following day. The CIS-R also provided data on 9 of the 12 associated symptoms of CFS listed in NICE guidelines,^39^ namely: muscle or joint pain, headaches, painful glands, sore throat, problems with memory or concentration (cognitive dysfunction), dizziness, nausea, and insomnia (as part of the ‘difficulty sleeping’ symptom in the NICE guideline). Adolescents without any of these accompanying symptoms were reclassified as non-CFS. Because data on only 9 of the 12 symptoms included in the NICE guideline could be collected using the CIS-R, the estimates of CFS at 17 years are likely to be conservative (ie, an adolescent may have been classified as non-CFS because of the lack of 1 of the 9 symptoms, but he/she may have had 1 of the other 3 symptoms for which data were uncollected). Of those identified as having CFS, 16.5% had a single symptom, 29.13% had 2, 25.54% had 3, 10.68% had 4, 8.74% had 5, 5.83% had 6, 2.91% had 7, and .97% had 8. Adolescents were classified as non-CFS if they reported having had problems with alcohol or drugs (crack, solvents, heroin, or cocaine) during the previous year, or a diagnosis of anorexia nervosa.

### Covariates

We chose measures known to be associated with either CFS or CWP.^7^, ^15^, ^17^, ^21^, ^22^, ^23^ Depression and anxiety were assessed using the CIS-R,^30^ a self-administered, computerized interview completed at age 17 at the research clinic. The CIS-R is adapted from the Clinical Interview Schedule to allow lay interviewers to assess psychiatric morbidity in the community. It is a valid instrument for the detection of a range of common mental disorders in various countries^31^, ^48^, ^49^ although in the United Kingdom, although demonstrating acceptable levels of specificity (.94, 95% confidence interval [CI] = .90–.97), levels of sensitivity are less favorable (.31; 95% CI = .09–.61).^9^

The CIS-R includes 14 sections establishing the severity of different symptom clusters: somatic symptoms, fatigue, concentration, sleep, irritability, worries over physical health, depression, depressive ideas, worry, anxiety, phobias, panic, compulsions, and obsessions. Initial filter questions in each section establish the existence of a particular symptom in the previous month, leading to a more detailed assessment focusing on the past week. For the assessment of anxiety and depression, we used the specific anxiety (5 questions) and depression (4 questions) subscales, both of which are scored from 0 (least severe) to 4 (most severe) depending on the symptom's frequency and severity. Height was measured to the last complete millimeter using a Harpenden stadiometer (Holtain Limited, Crymych, Dyfed, United Kingdom). Weight was measured to the nearest 50 g with a Tanita Body Fat Analyzer (Tanita Corp, Tokyo, Japan). Body mass index (BMI) was derived from clinic-measured height and weight (weight [kg]/height [m^2^]). Classifications of underweight, recommended weight, overweight, and obesity were produced in accordance with the International Obesity Task Force cutoffs.^14^ Mothers' highest educational qualification was used as a proxy for socioeconomic status.^26^ This was assessed at 32 weeks' gestation and categorized as none/minimal formal qualification, vocational qualification, O level (General Certificate of Education: Ordinary Level), A level (General Certificate of Education: Advanced Level), and university degree.

### Statistical Methods

Data used in the analyses were on the basis of those who had completed the pain questionnaire and CIS-R and had complete data for sex, maternal education, and BMI. The participants were categorized according to their pain/fatigue status; those with neither, those with exclusive CFS, those with exclusive CWP, and those with CWP as well as CFS. These categories were compared against the other variables using chi-squared testing (with Fisher exact test used where appropriate). The relationship between CFS and CWP with our chosen covariates was assessed using logistic regression, using a crude model and an adjusted model, to calculate odds ratios (ORs) and 95% CIs. In cases in which the covariate had more than 2 categories the appropriate use of a common OR was assessed using likelihood ratio testing.

The relationship between CFS and the severity of pain experienced was tested using ordered logistic regression in crude and adjusted models. Although the original pain rating variable was an ordinal 1 to 10 scale these data were collapsed into a 4-category outcome for ease of analysis; no pain (1 of 10), a score of 2 (5 of 10), a score of 6 (9 of 10), and a score of 10 (10 of 10). The proportional odds assumption was examined using a likelihood ratio test for each pain rating outcome. Finally the associations between pain/fatigue status and our covariates were examined using multinomial logistic regression, which provides a relative risk ratio (RRR) for each factor comparative to our reference category of neither CFS nor CWP. All statistical analyses were conducted using Stata 13.1 (Stata Corp, College Station, TX).

## Results

Of 13,978 children alive at 1 year, 5,217 attended the 17-year research clinic. Of these, 4,001 (76.7%) completed the pain questionnaire and 4,564 (87.5%) completed the CIS-R session. There were 3,214 adolescents (61.6%) with complete data for all outcomes and covariates. Of these individuals, 1,349 were male (42.0%). The average age of the participants at the time of clinic attendance was 17.8 years (SD = .4). Compared with the complete cohort, those included in the current study were more likely to have mothers educated to at least degree level (20.3% vs 10.2%) and who gave birth at a later age (29.4 years vs 27.5 years). Compared with those with complete data, those who only had pain data did not have different ratings of pain: at the current time, at its worst, or ratings of disruption of activities from pain. However, the sample with only pain data did have a higher proportion of adolescents reporting the highest rating of pain on average (2.15% vs 1.84%, *P* = .002). There were no differences in levels of fatigue, depressive, or anxiety symptoms in those with complete data versus those who only had data from the CIS-R session.

There were 82 individuals (2.6%) classified with CFS and 145 (4.5%) with CWP. There were 12 individuals (.4%) who were classified with both, corresponding to an overlap of 14.6% of those with CFS also having comorbid CWP.

Table 1 shows descriptive statistics for those with exclusive CFS and exclusive CWP on the basis of those with complete data for CFS, CWP, maternal education, and BMI (it was decided not to present data for those with CFS together with CWP because the sample was small and cross-tabulations with covariates could potentially lead to disclosure of participants). CFS and CWP were more common in female participants, despite only representing 58% of the sample; the proportion of those classified as CFS and CWP who were female was 74.3% and 72.9%, respectively. Those reporting moderate headaches had a higher prevalence of exclusive CFS and exclusive CWP, compared with those not reporting moderate headaches. The same was apparent for moderate abdominal pain. There were differences in the prevalence of the conditions across levels of anxiety and depression, with higher levels of anxiety or depression being associated with higher prevalence of the condition (except for depression and CWP). Data for those with CFS together with CWP are omitted from Table 1 because of low cell counts, which could potentially lead to issues of deductive disclosure. However, associations similar to those reported for exclusive CFS and exclusive CWP were observed for this group (ie, no association with maternal education or BMI category, but greater prevalence in those reporting moderate headaches, abdominal pain, and in those with higher levels of anxiety and depression).

### Associated Factors With CFS and CWP

#### CFS

Unadjusted estimates reveal that female participants had more than twice the risk of being classified as CFS (OR = 2.14, 95% CI = 1.30–3.53). A 1-unit increase in the depression and anxiety scales resulted in an increase in odds in the magnitude of 2.47 (95% CI = 2.21–2.86) and 2.50 (95% CI = 2.14–2.90), respectively.

Table 2 shows that the associations with anxiety (OR = 1.72, 95% CI = 1.42–2.08) and depression (OR = 1.87, 95% CI = 1.56–2.25) were attenuated after adjustment for obesity and maternal education.

#### CWP

In the unadjusted models, female participants had twice the odds receiving a classification of CWP compared with male participants (OR = 2.02, 95% CI = 1.39–2.94). A 1-unit increase in the depression and anxiety scales was once again associated with an increased odds of CWP (OR = 1.40, 95% CI = 1.22–1.62 and OR = 1.53, 95% CI = 1.32–1.78). Adjusting for obesity and maternal education resulted in a weakened association with anxiety and no association with depression.

The OR between CFS and CWP revealed that a classification of 1 (vs no classification) was associated with 387% higher odds of receiving a classification of the other (OR = 3.87, 95% CI = 2.05–7.31), such that those with a classification of CFS were approximately 4 times more likely to be classified as having CWP, compared with those not classified as CFS. However, after adjustment for the covariates, the strength of evidence for this association attenuated to the null (OR = 1.92, 95% CI = .91–4.06).

### Relationship Between CFS and CWP and the Experience of Pain

Table 3 shows the crude and adjusted associations between the presence of exclusive CFS and exclusive CWP and the ratings of pain (at its worst and on average), its interference in daily life, and its effect on disruption to social activities. Unadjusted estimates reveal that compared with not being classified as having exclusive CFS, those with exclusive CFS were more likely to report a higher category of average pain over the past 6 months (OR = 2.17, 95% CI = 1.21–3.92). The presence of exclusive CFS produced an OR of 3.50 (95% CI = 2.02–6.09) for reporting a higher category of pain-related daily interference, compared with not having exclusive CFS. Finally, compared with those without CFS, the OR of reporting a higher category of disruption to social activities as a result of their pain was 2.23 (95% CI = 1.28–3.88) for those who were classified as having exclusive CFS. After adjustment for covariates, only the association between exclusive CFS and daily interference as a result of pain remained (OR = 2.19, 95% CI = 1.24–3.88).

Unadjusted estimates in those with exclusive CWP (vs not) were more than 2.5 times more likely to report a higher category of pain when at its most intense (OR = 2.58, 95% CI = 1.78–3.74), with a similar likelihood to report a higher category of average pain. A higher level of pain- related daily interference (OR = 1.84, 95% CI = 1.30–2.60) and a higher category of disruption to social activities as a result of their pain (OR = 1.58, 95% CI = 1.13–2.22), was also reported in those with exclusive CWP; interestingly, these estimates were lower than those reported in those with exclusive CFS. These associations remained with minimal attenuation after adjustment for the covariates.

### Associated Factors With Exclusive CFS, Exclusive CWP, and Comorbid CFS and CWP

Table 4 describes the relationship between exclusive CFS, exclusive CWP, the 2 conditions occurring together, and obesity, maternal education, depression, and anxiety (all relative to having neither of the conditions). As the RRRs reveal, a 1-unit increase in the depression and anxiety scales was associated with greater risk of being classified as having exclusive CFS, compared with neither CFS or CWP (RRR = 1.83, 95% CI = 1.50–2.22; RRR = 1.75, 95% CI = 1.43–2.15, respectively), with the other variables in the model held constant. Female sex (relative to male sex) was associated with a greater likelihood of being classified as having exclusive CWP, as opposed to having neither of the conditions (RRR = 1.87; 95% CI = 1.26–2.77). This was also true for increasing levels of anxiety (RRR = 1.34, 95% CI = 1.10–1.63), but with no association between depression and exclusive CWP. Increasing levels of depression and anxiety resulted in a greater likelihood of being classified as having comorbid CFS and CWP relative to having neither of the conditions (RRR = 2.41, 95% CI = 1.51–3.84; RRR = 2.04, 95% CI = 1.30–3.21, respectively).

## Discussion

### Summary of Findings

To our knowledge, this is the first population-based study to present prevalence rates of CFS, CWP, and the prevalence of overlap between these 2 conditions in adolescence. In addition, we examined similarities and differences in pain variables among these conditions, as well as covariates such as obesity, depression, and anxiety.

In this cohort, 14.6% of those with CFS also experienced CWP. Unsurprisingly therefore, the presence of 1 of the conditions had a strong predictive effect for the presence of the other condition (OR = 3.87, 95% CI = 2.05–7.31). Female participants were approximately twice as likely to have CFS or CWP, although this sex effect was attenuated after adjustment for obesity and maternal education and CWP or CFS. Those with CFS were more likely to report a higher level of pain and greater effect of such pain compared with those without CFS. Multinomial regression revealed that relative to having neither CFS nor CWP, a 1-unit increase in the depression and anxiety scales increased the risk of having exclusive CFS and, to a greater extent, the risk of having comorbid CFS and CWP, whereas only anxiety (not depression) showed an association with exclusive CWP. Female participants had a greater risk of having exclusive CWP (relative to having neither) and interestingly, this risk was greater and supported more strongly than for the female risk for exclusive CFS and both conditions co-occurring. We did not find an association between obesity or maternal education with either exclusive CFS or CWP.

### Comparison With Other Studies

In a study of adolescents with CFS versus healthy control participants,^68^ it was reported that, similar to this study, adolescents with CFS had higher self-reported pain severity and pain interference scores. However, unlike in this study, adjustment for depression and anxiety was not made and thus the authors were unable to rule out the likely influence of confounding on these associations. It has been hypothesized that the increased sensitivity to pain in those with CFS^51^, ^53^ could be a result of a general state of hypersensitivity^33^ with a resulting hyperalgesia phenotype.^34^ For example, those with CFS have also been reported to have lower thresholds to thermal and electrical stimuli.^60^, ^62^ This central sensitization hypothesis has also been implicated in the pathogenesis of CWP^33^ and fibromyalgia.^69^ Interestingly, when comparing the ratings of pain and interference in those with exclusive CFS versus exclusive CWP, we observed no differences, however, as Table 3 shows, all of the associations between the presence of CWP and ratings of pain and interference remained after adjustment for covariates, whereas in the case of exclusive CFS, only the association with daily interference remained after adjustment, which may suggest that these covariates play a greater role in the manifestation of pain in those with CFS versus CWP.

In this study, although higher levels of depression and anxiety scores remained associated with exclusive CFS, only anxiety appeared to be related to exclusive CWP. Although depression and anxiety have been strongly and consistently associated with CFS and fibromyalgia, most study designs have made it impossible to separate the possible influence of an overlapping CFS/fibromyalgia comorbidity. Indeed the results from the multinomial regression are in contrast to the results from the logistic regression in which CFS and CWP were not separated, and which resulted in the presence of an association between CWP and depression scores (Table 2). It can be speculated therefore, that this association was actually driven by the relationship between depression and the comorbid CFS. In our study, increasing levels of anxiety and depression were more strongly associated with the presence of CFS and CWP together, than the 2 conditions independently, which may be a consequence of the greater morbidity.

Female participants were more likely to be classified as CFS, which is in line with previous estimates in this cohort, at this age (Norris, 2016 unpublished data) and earlier in adolescence.^15^ We also observed an increased risk of exclusive CWP in female compared with male participants, which is in line with previous reports of the condition^13^ and of fibromyalgia.^2^, ^22^, ^27^, ^32^, ^69^ It is of interest that this female-associated risk for exclusive CWP was of greater severity and strength than for exclusive CFS. This greater disparity in the female:male ratio observed for CWP has also been observed for fibromyalgia. However, it has been speculated that this sex disparity in fibromyalgia is a consequence of the previous American College of Rheumatology criteria,^68^, ^70^ which required the presence of 11 of 18 tender points, a finding that occurs much more commonly in women.^66^, ^69^ Because the tender points criteria is not included in the CWP diagnosis, this phenomenon cannot be explaining the increased prevalence of CWP in female compared to male participants. We are not certain as to the reason behind this increased prevalence of CWP in female participants, but studies have attributed the increased risk observed in women to various factors, including the differential effects of the gonadal hormones,^2^, ^3^, ^20^, ^45^ differences in endogenous pain modulation,^19^, ^24^ and divergent ‘gender’ roles.^6^, ^36^

In this study, obesity was found to have little effect on either exclusive CFS or CWP. The lack of an effect of obesity on CWP has been observed previously in this cohort, with Deere et al reporting no effect of obesity on the presence of CWP but using a different classification of obesity.^23^ However, they did find an association between obesity and other pain phenotypes. Studies investigating the relationship between obesity and CFS have also reported a lack of association in young people^63^ and adults.^12^ The current study is unique, however, in that, unlike the previous studies, the findings are for the exclusive conditions of CWP or CFS, and thus the effect of obesity can be investigated without the issue of overlap between the 2 conditions potentially affecting any association (or lack of association).

### Strengths and Limitations

To the authors’ knowledge, this is the first study to investigate differences in a range of covariates between adolescents with exclusive CFS, exclusive CWP, or both morbidities coexisting. One of the limitations of this study is that the 3,214 adolescents included represent only a minority of the approximately 14,000 mother-child dyads who were originally enrolled in the study, resulting in a selected study sample. For example, compared with the complete cohort, those included in the current study were more likely to have mothers educated to at least degree level (20.3% vs 10.2%) and who gave birth at a later age (29.4 years vs 27.5 years). Nonetheless, we have no reason to believe that the association between pain and CFS differs according to maternal age and/or educational status and thus we doubt whether this would have influenced the results. However, because of the relative infrequency of both conditions, the number of participants with CFS together with CWP provided a very small sample (n = 12) from which to draw conclusions. The fact that the study was questionnaire-based means that there is a possibility that recall bias was introduced into the results. For example, the strong association between depression and CFS may influence the reporting of pain and its severity. However, associations persisted after adjustment for depression (Table 3). Furthermore, the retrospective nature of the questionnaire (relating to experiences of pain experienced at least 3 months ago) means that the passing of time could introduce further bias into pain ratings. For example, it has been observed that when retrospectively rating experiences of average pain, recall is found to be a combination of ratings of pain at its peak and at the end of the experience, although the effect was small.^28^, ^57^ A further limitation is that because data on only 9 of the 12 symptoms included in the NICE guideline could be collected using the CIS-R, the estimates of CFS at 17 years may be underestimated. Finally, the sensitivity analysis of those with only pain data versus those with complete data shows we may have introduced some selection bias into our sample, because the sample with only pain data did show a higher proportion of adolescents reporting more severe levels of pain on average, compared with those with complete data, however there were no differences in the other pain ratings, fatigue, depression, and anxiety.

Apart from sex, we do not know whether the associations we have described are causal or secondary to the CFS and CWP,^7^ because these characteristics were all collected at the same point in time (ie, a cross-sectional study). Although longitudinal studies in children and adolescents have suggested that the direction of causation might be that anxiety and depression lead to fatigue,^58^, ^64^ it is uncertain whether this can be extended to CFS.^7^ For example, a recent study in the same cohort showed that after adjustment for maternal psychopathology, childhood mood problems were not predictive of CFS at 13 years.^17^

Because the pain questionnaire administered did not include questions relating to areas of tenderness, we were unable to classify adolescents as having fibromyalgia and are thus unable to draw any specific conclusions about the relationship between CFS and fibromyalgia in this population. Because CWP is the primary symptom of fibromyalgia,^38^ it may however be speculated that similar relationships may have been observed, had it been possible to classify this condition. Nonetheless, further research is required to identify whether the relationships observed with CWP in this study are maintained in cases of fibromyalgia.

This study showed that 14.6% of adolescents with CFS have comorbid CWP, and the likely greater proportion of more mild cases observed in this epidemiological study means that the prevalence of overlap is likely to be underestimated compared with those attending specialist services. Because severe pain is associated with a worse outcome in adults,^11^, ^29^ clinicians should be aware of the overlap between these 2 conditions and should carefully consider the treatment options offered. Developing appropriate, targeted interventions for children with CFS and chronic pain may increase the likelihood of improved outcomes.

## Conclusions

Future research should investigate whether depression and anxiety are causal or secondary to CFS and whether anxiety is causal for chronic pain syndrome. Furthermore, it is important to elucidate the factors that are contributing to the higher prevalence of CFS, and to a greater degree, CWP, observed in women.

## Figures and Tables

**Table 1:** Descriptive Statistics

Covariate	n	Exclusive CFS (n = 70)		Exclusive CWP (n = 133)	
		n (%)	*P*	n (%)	*P*
Sex					
Male	1349	18 (1.3)	.005	36 (2.7)	<.001
Female	1865	52 (2.8)		97 (5.2)	
Maternal education					
CSE or vocational	593	9 (1.52)	.191	26 (4.38)	.768
O level	1071	25 (2.3)		43 (4.0)	
A level	899	26 (2.9)		41 (4.6)	
Degree	651	10 (1.5)		23 (3.5)	
BMI category (IOTF)					
Underweight	242	8 (3.0)	.282	6 (2.5)	.306
Recommended	2256	51 (2.3)		92 (4.0)	
Overweight	502	6 (1.2)		22 (4.4)	
Obese	214	5 (2.4)		13 (6.1)	
At least moderate headaches					
No	2748	45 (1.6)	<.001	89 (3.2)	<.001
Yes	466	25 (5.4)		44 (9.4)	
At least moderate abdominal pain					
No	2840	48 (1.7)	<.001	79 (2.8)	<.001
Yes	374	22 (5.9)		54 (14.4)	
Anxiety score					
0	2698	28 (1.0)	<.001	99 (3.7)	.006
1	273	12 (4.4)		14 (5.1)	
2	132	11 (8.3)		11 (8.3)	
≥3	111	19 (17.12)		9 (8.11)	
Depression score					
0	2539	22 (.9)	<.001	95 (3.7)	.108
1	354	15 (4.2)		17 (4.8)	
2	169	8 (4.7)		11 (6.5)	
≥3	152	25 (16.45)		10 (6.58)	

Abbreviations: CSE, Certificate of Secondary Education; O level, General Certificate of Education: Ordinary Level; A level, General Certificate of Education: Advanced Level; IOTF, International Obesity Task Force.

NOTE. N on the basis of complete data availability for pain, fatigue, and covariates. *P* on the basis of χ^2^ test for comparison of CFS and CWP (separately), with the total sample.

**Table 2:** Logistic Regression Showing Odds of CFS and CWP

Outcome	Covariate	Crude			Multivariable		
		OR	95% CI	*P*	OR	95% CI	*P*
CFS	CWP versus no CWP	3.87	2.05–7.31	<.001	1.92	.91–4.06	.086
	Female versus male	2.14	1.30–3.53	.003	1.29	.75–2.21	.353
	Obesity versus non-obese	.91	.36–2.27	.837	.82	.32–2.15	.682
	Maternal education	1.07	.89–1.29	.445	1.12	.92–1.37	.262
	Depression score	2.47	2.12–2.86	<.001	1.87	1.56–2.25	<.001
	Anxiety score	2.50	2.14–2.90	<.001	1.72	1.42–2.08	<.001
CWP	CFS versus no CFS	3.87	2.05–7.31	<.001	1.98	.97–4.04	.059
	Female versus male	2.02	1.39–2.94	<.001	1.79	1.22–2.62	.003
	Obesity versus nonobese	1.41	.78–2.53	.256	1.35	.74–2.46	.327
	Maternal education	1.00	.88–1.15	.951	1.03	.89–1.18	.712
	Depression score	1.40	1.22–1.62	<.001	1.14	.95–1.36	.155
	Anxiety score	1.53	1.32–1.78	<.001	1.32	1.10–1.58	.003

NOTE. N = 3214. OR represents odds of outcome per unit increase in covariate. Adjusted model includes adjustment for sex, obesity, maternal education, depression score, anxiety score, and mutual adjustment for CWP/CFS.

**Table 3:** Ordered Logistic Regression Examining the Relationship Between CFS and Rating of Pain

	Pain Rating in Past 6 Mo	n	Crude			Multivariable		
			OR	95% CI	*P*	OR	95% CI	*P*
Exclusive CFS	At worst	1483	1.82	.99–3.35	.055	1.18	.33–2.23	.599
	On average	1469	2.17	1.21–3.92	.010	1.49	.80–2.80	.204
	Daily interference	1533	3.50	2.02–6.09	<.001	2.19	1.24–3.88	.007
	Disruption to social activities	1501	2.23	1.28–3.88	.004	1.40	.79–2.48	.247
Exclusive CWP	At worst	1483	2.58	1.78–3.74	<.001	2.39	1.65–3.48	<.001
	On average	1469	2.42	1.70–3.44	<.001	2.23	1.55–3.21	<.001
	Daily interference	1533	1.84	1.30–2.60	.010	1.72	1.21–2.44	.001
	Disruption to social activities	1501	1.58	1.13–2.22	.008	1.53	1.06–2.12	.021

NOTE. n indicates those with complete data for all covariates. Adjusted model adjusted for sex, obesity, maternal education, anxiety score and depression score. OR represents odds of those with exclusive CFS or exclusive CWP being in a higher category of pain rating than those without CFS or CWP, respectively.

**Table 4:** Multinomial Regression Examining the Relationship Between CWP and CFS

Outcome	Covariate	RRR	95% CI	*P*
Neither∗	—	Reference category		
Exclusive CFS	Female (vs male)	1.44	.81–2.54	.212
	Obesity (vs nonobese)	.95	.36–2.50	.910
	Maternal education	1.09	.88–1.35	.413
	Depression	1.83	1.50–2.22	<.001
	Anxiety	1.75	1.43–2.15	<.001
Exclusive CWP	Female (vs male)	1.87	1.26–2.77	.002
	Obesity (vs nonobese)	1.46	.80–2.66	.218
	Maternal education	1.01	.87–1.17	.907
	Depression	1.11	.92–1.35	.279
	Anxiety	1.34	1.10–1.63	.004
Both†	Female (vs male)	1.12	.28–4.47	.873
	Obesity (vs nonobese)	—	—	—
	Maternal education	1.50	.86–2.61	.153
	Depression	2.41	1.51–3.84	<.001
	Anxiety	2.04	1.30–3.21	.002

NOTE. N = 3214. For RRR, for a 1-unit increase in covariate comparative to the reference group, calculated by exponentiating the relative log odds. Results blank where there were no cases of obesity.

∗ Neither represents those that had neither CWP nor CFS.

† Individuals who had CWP and chronic disabling fatigue.
