# Supplementary material for: Chronic Fatigue Syndrome and Chronic Widespread Pain in Adolescence: Population Birth Cohort Study
Source: J Pain. 2017 Mar;18(3):285–94. doi: 10.1016/j.jpain.2016.10.016 (PMC5340566; doi:10.1016/j.jpain.2016.10.016)
Supplement: Supplementary File 1 [file mmc1.docx]

**Supplementary file 1: Diagnostic criteria for Chronic Widespread Pain (CWP), fibromyalgia and Chronic Fatigue Syndrome (CFS)**

CWP and fibromyalgia: The American College of Rheumatology 1990 criteria (Wolfe et al. 1990)

| 1. **History of widespread pain** |
| --- |
| Definition: Pain is considered widespread when all of the following are present: pain in the left side of the body, pain in the right side of the body, pain above the waist, pain below the waist. In addition, axial skeletal pain (cervical spine or anterior chest or thoracic spine or low back) must be present. In this definition, shoulder and buttock pain is considered as pain for each involved side. “Low back” pain is considered lower segment pain. |
| 1. **Pain in 11 of 18 tender point sites on digital palpation** |
| Definition: Pain, on digital palpation, must be present in at least 11 of the following 18 tender point sites:  Occiput: bilateral, at the sub-occipital muscle insertions  Low cervical: bilateral, at the anterior aspects of the inter-transverse spaces at c5-c7  Trapezius: bilateral, at the midpoint of the upper border  Supraspinatus: bilateral, at origins, above the scapula spine near the medial border  Second rib: bilateral, at the second costochondral junctions, just lateral to the junctions on upper surfaces  Lateral epicondyle: bilateral, 2cm distal to the epicondyles  Gluteal: bilateral, in upper outer quadrants of buttocks in anterior fold of muscle  Greater trochanter: bilateral, posterior to the trochanteric prominence  Knee: bilateral, at the medial fat pad proximal to the joint line  Digital palpation should be performed with an approximate force of 4kg  For a tender point to be considered “positive” the subject must state that the palpation was painful. “Tender” is not to considered “painful” |

^For classification purposes, patients will be said to have fibromyalgia if both criteria are satisfied. Widespread pain must have been present for at least 3 months, to be classified as chronic (CWP)^

CFS: National Institute of Health and Care Excellence criteria (NICE 2007)

| 1. **Fatigue with all of the following features:**   New or had a specific onset (that is, it is not lifelong)  Persistent and/or recurrent  Unexplained by other conditions  Has resulted in a substantial reduction in activity level  Characterised by post-exertional malaise and/or fatigue (typically delayed, for example by at least 24 hours, with slow recovery over several days)  Which has persisted for:  4 months in an adult  3 months in a child or young person; the diagnosis should be made or confirmed by a paediatrician |
| --- |
| 1. **One or more of the following symptoms:**   Difficulty with sleeping, such as insomnia, hypersomnia, unrefreshing sleep, a disturbed sleep-wake cycle  Muscle and/or joint pain that is multi-site and without evidence of inflammation  Headaches  Painful lymph nodes with pathological enlargement  Sore throat  Cognitive dysfunction, such as difficulty thinking, inability to concentrate, impairment of short-term memory, and difficulties with word-finding, planning/organising thoughts and information processing  Physical or mental exertion makes symptoms worse  General malaise of ‘flu-like’ symptoms  Dizziness and/or nausea  Palpitations in the absence of identified cardia pathology |

CFS: Centers for Disease Control and Prevention criteria (Fukuda 1994)

| 1. **Fatigue with all of the following features:**   Persistent and/or relapsing  New or had a specific onset (has not been lifelong)  Not the result of ongoing exertion  Not substantially alleviated by rest  Has resulted in a substantial reduction in previous levels of occupational, educational, social or person activities  Which has persisted for:  6 or more consecutive months |
| --- |
| 1. **At least 4 of the following symptoms:**   Impaired memory or concentration  Sore throat  Tender cervical or axillary lymph nodes  Muscle pain  Multi-joint pain  New headaches  Unrefreshing sleep  Post-exertional malaise |
